# Identification of a Porcine Liver Eomes^high^T-bet^low^ NK Cell Subset That Resembles Human Liver Resident NK Cells

**DOI:** 10.3389/fimmu.2019.02561

**Published:** 2019-10-31

**Authors:** Steffi De Pelsmaeker, Sofie Denaeghel, Leen Hermans, Herman W. Favoreel

**Affiliations:** Laboratory of Immunology, Department of Virology, Parasitology and Immunology, Faculty of Veterinary Medicine, Ghent University, Merelbeke, Belgium

**Keywords:** natural killer cells, NK cells, tissue residency, Eomes, T-bet, liver, pig

## Abstract

Natural killer (NK) cells are cells of the innate immunity and play an important role in the defense against viral infections and cancer, but also contribute to shaping adaptive immune responses. Long-lived tissue-resident NK cells have been described in man and mouse, particularly in the liver, contributing to the idea that the functional palette of NK cells may be broader than originally thought, and may include memory-like responses and maintaining tissue homeostasis. Remarkably, liver resident (lr)NK cells in man and mouse show substantial species-specific differences, in particular reverse expression patterns of the T-box transcription factors Eomesodermin (Eomes) and T-bet (Eomes^high^T-bet^low^ in man and *vice versa* in mouse). In pig, compared to blood NK cells which are CD3^−^CD8α^high^ cells, the porcine liver contains an abundant additional CD3^−^CD8α^dim^ NK cell subpopulation. In the current study, we show that this porcine CD3^−^CD8α^dim^ liver NK population is highly similar to its human lrNK counterpart and therefore different from mouse lrNK cells. Like human lrNK cells, this porcine NK cell population shows an Eomes^high^T-bet^low^ expression pattern. In addition, like its human counterpart, the porcine liver NK population is CD49e^−^ and CXCR6^+^. Furthermore, the porcine Eomes^high^T-bet^low^ liver NK cell population is able to produce IFN-γ upon IL-2/12/18 stimulation but lacks the ability to kill K562 or pseudorabies virus-infected target cells, although limited degranulation could be observed upon incubation with K562 cells or upon CD16 crosslinking. All together, these results show that porcine Eomes^high^T-bet^low^ NK cells in the liver strongly resemble human lrNK cells, and therefore indicate that the pig may represent a unique model to study the function of these lrNK cells in health and disease.

## Introduction

Natural killer (NK) cells are important members of the innate immune system and play a crucial role in the defense against viral infections and cancer, mainly because of their ability to kill virus-infected cells and malignant cells. In addition, NK cells may also produce different cytokines, particularly interferon gamma (IFN-γ), thereby steering the adaptive immune response toward a Th1 response (1–3). Although NK cells have traditionally been considered to be short-lived, increasing evidence in man and mouse indicates that long-lived NK cell subsets exist, particularly in the liver (4). Such long-lived NK cell subpopulations contribute to the notion that the functional repertoire of NK cells may be broader than originally thought, e.g., including memory-like responses and maintenance of tissue homeostasis (4, 5). Although both human and mouse livers harbor long-lived tissue resident NK cells, these liver resident NK cells (lrNK cells) show remarkable differences in phenotype between both species, including in their expression of T-box transcription factors. Indeed, human lrNK cells are characterized by a strong expression of the T-box transcription factor Eomesodermin (Eomes) and low expression of T-bet, whereas murine lrNK cells show the exact opposite T-box transcription factor expression pattern (6–9). In addition, in mouse, CD49a has been considered a defining cell surface marker for lrNK cells, whereas the human Eomes^high^ lrNK population does not overlap with the CD49a^+^ population (9, 10). Recently, however, CyTOF analysis revealed that lack of CD49e expression is a discriminating marker for human lrNK cells (7). Given these discrepancies between human and murine lrNK cells, additional/alternative animal model species may be useful to study these particular aspects of human NK cell biology. It has been shown that several elements of the pig immune system, including particular aspects of NK cells, closely relate to that of man (11–16). Previous studies showed that, compared to blood NK cells, the porcine liver contains an abundant additional subpopulation of NK cells that is characterized by a CD3^−^CD8α^dim^NKp46^high^CD16^+^ expression pattern (17, 18). It is currently unclear if and to what extent the CD8α^dim^NKp46^high^ NK cell population in pig may be phenotypically related to human and/or mouse lrNK cells. Therefore, in this study, we investigated whether porcine NK cells remaining in the porcine liver vasculature after perfusion display features that relate to human (and/or mouse) lrNK cells. Our data demonstrate that this porcine liver NK cell population is phenotypically remarkably similar to the human lrNK cell population and therefore different from mouse lrNK cells. Hence, the pig represents an attractive model to study the function of these lrNK cells in health and disease.

## Materials and Methods

### Primary Porcine Blood NK Cell Isolation and Culture

Heparinised blood samples (50 units/ml blood, LEO Pharma) were obtained from the external jugular vein of pigs (6-month-old pigs or 5-week-old piglets, crossbred pigs derived from Rattlerow-Segher hybrid sows and Pietrain boar) that were kept at the Faculty of Veterinary Medicine. The blood sampling and euthanasia procedures were approved by the Ethical Committee of the Faculty of Veterinary Medicine (EC2013/62 and EC2017/121). Primary porcine NK cells were isolated from porcine PBMC as described before (12, 13), by MACS depletion of CD3^+^ and CD172a^+^ cells followed by FACS purification based on their CD3^−^CD172a^−^CD8α^+^ expression profile using a BD FACS Aria III Cell Sorter (BD Biosciences), resulting in a 98.62 ± 0.50% pure porcine NK population. Hybridomas for antibodies against porcine CD172a [IgG_1_, clone 74-22-15a, (19)], CD3 [IgG_1_, clone PPT3, (20)], CD8α [IgG_2a_, clone 11/295/33, (21)] were kindly provided by Dr. A. Saalmüller (University of Veterinary Medicine, Vienna, Austria) and antibody stocks were produced in-house.

Primary porcine NK cells were cultured in 96-well flat bottomed plates (Nunc, Thermo Fisher Scientific) at a density of 5 × 10^6^ cells/ml in RPMI (Gibco), supplemented with 10% (v/v) fetal calf serum (Thermo Fisher Scientific), 100 U/ml penicillin (Gibco), 100 μg/ml streptomycin (Gibco) (referred to as porcine NK medium). For cytolytic assays, porcine NK cells were primed with recombinant human (rh) interleukin 2 (IL-2) (20 ng/ml) (Thermo Fisher Scientific) or with rh IL-2 (20 ng/ml), recombinant porcine (rp) IL-12 (25 ng/ml) (R&D Systems) and rp IL-18 (100 ng/ml) (R&D Systems) for 16–18 h before the assay.

For the plasticity experiment, porcine NK cells were primed with rh IL-2 (20 ng/ml), rp IL-12 (25 ng/ml), rp IL-15 (15 ng/ml) (Kingfisher Biotech), and/or rp IL-18 (100 ng/ml). Cells were cultured for 4 days with a 75% medium change at day 2.

### Isolation of Porcine Liver NK Cells

After dissection of the liver, porcine liver NK cells were isolated following a procedure adapted from earlier studies on human liver NK cells (7). The portal vein was perfused with 5 l ice-cold PBS (6-month old pigs) or with 1 l ice-cold PBS (5-week old piglets). A further 700 ml PBS was used for a final flush that was collected in different centrifuge tubes, followed by concentration of the perfusate by centrifugation. The resulting cell pellet was resuspended in PBS (room temperature) followed by the same protocol as described above for isolation of primary porcine blood NK cell isolation. Purification of liver NK subsets was done by FACS using a BD FACS Aria III Cell Sorter, essentially as described above for the isolation of porcine blood NK cells. FACS purification resulted in a 97.1 ± 1.42% pure population of porcine CD3^−^CD172a^−^CD8α^+^ liver NK cells and in a 94.93 ± 2.16% pure population of porcine CD3^−^CD172a^−^CD8α^dim^ liver NK cells.

### Flow Cytometric Analysis of Cell Surface Markers and Transcription Factors

For flow cytometric analysis of cell surface markers, cells were washed in PBS. All incubation steps were performed in 96-well conical bottomed plates for 30 min at 4°C. The different combinations of primary monoclonal antibodies (mAbs) and secondary reagents used for each assay are listed in Table 1 and antibodies were diluted in PBS. The anti-CD49e VC5 antibody (BD Biosciences) that was used reacts with CD49e of pig. The anti-human T-bet and Eomes antibodies that were used have been reported before to crossreact with porcine T-bet and Eomes, respectively (23, 24). The polyclonal anti-human CXCR6 antibody PA5-33461 (Thermo Fisher Scientific) is predicted to crossreact with porcine CXCR6 because the peptide used for generation of the antibody shows 94% amino acid identity between both species. Propidium iodide (Thermo Fisher Scientific) was used to discriminate live and dead cells for non-fixed cells stained for surface markers. Since freshly isolated cells consistently showed viability >96% no additional live/dead staining was performed for Eomes and T-bet staining where cells are fixed and permeabilized. For monoclonal antibody stainings, purified mouse IgG_1_ was used as an isotype control. For the polyclonal antibody stainings, control stainings were performed using only secondary antibody. For analysis of transcription factors, cells were fixed and permeabilized using the Foxp3/transcription factor staining buffer set (Thermo Fisher Scientific), according to the manufacturer's instructions. Next, cells were incubated with fluorescently labeled antibodies against the corresponding transcription factors (Table 1) at 4°C for 30 min and washed two times with Permeabilization buffer (Thermo Fisher Scientific). Purified mouse IgG_1_ (phycoerythrin conjugated) was used as an isotype control. To assess cell viability after 4 days culture in the plasticity experiment, a live/dead fixable violet dead cell stain kit (Invitrogen) was used before staining of cells for the transcription factors. Flow cytometry was performed using a NovoCyte Flow Cytometer (ACEA Biosciences), and samples were analyzed with NovoExpress software (ACEA Biosciences).

**Table 1:** Primary and secondary antibodies used for cell surface expression and transcription factor analysis by flow cytometry.

**Antigen**	**Clone**	**Isotype/recombinant**	**Labeling strategy**
**ANTIBODIES USED FOR PORCINE NK CELL SORTING**			
CD3 (20) Absent on NK cells	PPT3	IgG_1_	Secondary antibody R-phycoerythrin conjugated goat anti-mouse IgG_1_
CD172a (19) Absent on NK cells	74-22-15a	IgG_1_	Secondary antibody R-phycoerythrin conjugated goat anti-mouse IgG_1_
CD8α (21) Present on NK cells	11/295/33	IgG_2a_	Biotin-streptavidin Allophycocyanin Or secondary antibody Alexa fluor 647 goat anti-mouse IgG_2a_
**ANTIBODIES USED TO ANALYZE EXPRESSION OF CELL SURFACE MARKERS ON SORTED PORCINE NK CELLS**			
CD16 (Bio Rad)	G7	IgG_1_	Secondary antibody R-phycoerythrin conjugated goat anti-mouse IgG_1_
CD27 (Bio Rad)	B30C7	IgG_1_	Secondary antibody R-phycoerythrin conjugated goat anti-mouse IgG_1_
NKp46 (17)	VIV-KM1	IgG_1_	Secondary antibody R-phycoerythrin conjugated goat anti-mouse IgG_1_
CD49e (BD Biosciences)	VC5 Anti-human CD49e Crossreactive antibody	IgG_1_	Secondary antibody R-phycoerythrin conjugated goat anti-mouse IgG_1_
CXCR6 (Thermo Fisher Scientific)	Polyclonal Anti-human CXCR6 Crossreactive antibody	IgG	Secondary antibody R-phycoerythrin conjugated goat anti-rabbit IgG
CD25(22)	K231.3B2	IgG_1_	Secondary antibody R-phycoerythrin conjugated goat anti-mouse IgG_1_
**ANTIBODIES USED TO ANALYZE EXPRESSION OF T-BOX TRANSCRIPTION FACTORS IN SORTED PORCINE NK CELLS**			
T-bet (Thermo Fisher Scientific)	4B10 Anti-human T-bet Crossreactive antibody	IgG_1_	Directly labeled phycoerythrin conjugated
Eomes (Thermo Fisher Scientific)	WD1928 Anti-human Eomes Crossreactive antibody	IgG_1_	Directly labeled phycoerythrin conjugated

### Cytolytic Assays

K562 cells, mock-infected swine kidney (SK) cells and pseudorabies virus (PRV)-infected SK cells were used as target cells as described before (12, 13, 25–27). K562 cells were cultivated in IMDM (Life Technologies, Thermo Fisher Scientific) supplemented with 10% (v/v) FCS, 100 U/ml penicillin, 100 μg/ml streptomycin and 0.05 mg/ml gentamycin. SK cells were cultivated in suspension in MEM (Life Technologies, Thermo Fisher Scientific) supplemented with 10% (v/v) FCS, 100 U/ml penicillin, 100 μg/ml streptomycin and 0.05 mg/ml gentamycin and mock-inoculated or inoculated with PRV strain Kaplan at a multiplicity of infection (MOI) of 10 for 12 h. Target cells were labeled with carboxyfluorescein succinimidyl ester (CFSE) dye (Invitrogen, Thermo Fisher Scientific) according to the manufacturer's recommendations. Briefly, 1.0 × 10^6^ cells/ml were resuspended in porcine NK medium with 5 μM CFSE and incubated during 15 min at 37°C. The labeling reaction was stopped by addition of ice-cold porcine NK medium. Afterwards, cells were washed in porcine NK medium to remove excess CFSE. Target cells were co-incubated with IL-2 or IL-2/12/18-primed NK cells at an effector:target ratio of 20:1 for 4 h at 37°C. After co-incubation, viability of target cells was assessed by propidium iodide staining and flow cytometric analysis. The percentage of NK mediated lysis was calculated using the formula (% dead target_NK_-% dead target_spont_)/(%dead target_max_-% dead target_spont_).

### Degranulation Assays

Anti-CD16-induced degranulation assays were performed as described before (18). In brief, monoclonal antibodies against CD16 (IgG_1_, clone G7, Bio-Rad) were coated on 96-well round-bottom plates by overnight incubation at 4°C at a concentration of 6 μg/ml in PBS in a total volume of 50 μl per well. Isotype matched irrelevant antibodies (MG100, Thermo Fisher Scientific) at the same concentration served as control. Plates were washed with PBS three times before cells were added. Freshly isolated blood and liver porcine NK cells were primed with rh IL-2 (20 ng/ml) or with rh IL-2 (20 ng/ml), rp IL-12 (25 ng/ml), and rp IL-18 (100 ng/ml) for 16 h with 1 × 10^5^ cells in a total volume of 200 μl porcine NK medium per well, using 96-well round-bottom plates. After priming, cells were added to the coated plates. For K562-induced degranulation assays, K562 cells were co-incubated with primed NK cells at an effector:target ration of 1:1 in PBS coated 96-well round-bottom plates. Microcultures were supplemented with FITC-conjugated anti-CD107a mAb (IgG_1_, clone 4E9/11 Bio-rad, Serotec) at a final concentration of 4 μg/ml and two protein transport inhibitors Brefeldin A (GolgiPlug,BD Biosciences, final concentration 1 μg/ml) and Monensin (GolgiStop, BD Biosciences, final concentration 2 μg/ml) in a total volume of 200 μl porcine NK medium. After an incubation period of 4 h at 37°C, plates were centrifuged (5 min, 400 g, 4°C), cells were washed with 200 μl PBS and centrifuged again (5 min, 400 g, 4°C°). Cells were resuspended in 100 μl PBS with live-dead marker 7-Aminoactinomycin D (7-AAD, Invitrogen, Thermo Fisher Scientific) and analyzed by flow cytometry.

### IFN-γ ELISpot Assays

IFN-γ ELISpot was performed as described before (28). Briefly, 96-well MultiScreen-IP Filter Plates (0.45 μm, clear, sterile; Merck Millipore, US) were coated with mouse anti-porcine IFN-γ capture mAb (clone A151D5B8, Thermo Fisher Scientific; 10 μg/ml in PBS) overnight at 4°C. After blocking with pNK medium for 2 h at 37°C, 1.25 × 10^4^ NK cells were added per well in the presence or absence of cytokines. Cytokine mixes consisted of rh IL-2 (20 ng/ml), rp IL-12 (25 ng/ml), and/or rp IL-18 (100 ng/ml). Cells were incubated for 20 h or 38 h at 37°C. Thereafter, plates were washed with PBS/0.1% Tween-20 and plate-bound IFN-γ was visualized by incubation with a biotinylated mouse anti-swine IFN-γ mAb [clone A151D13C5, Thermo Fisher Scientific; 2.5 μg/ml in PBS/0.1% Tween20 with 0.1% bovine serum albumin (BSA, Thermo Fisher Scientific)] for 1 h at room temperature, followed by incubation with Streptavidin-alkaline phosphatase (1:2000 in PBS/0.1% Tween20 with 0.1% BSA) for 1 h at room temperature. Thereafter, SIGMAFAST1 BCIP/NBT substrate (Sigma–Aldrich) was used according to manufacturer's instructions. After intensive washing with ultrapure water, plates were allowed to dry thoroughly at 4°C and spots were analyzed on an Immunospot reader (Cellular Technology Ltd. Europe GmbH) and ImmunoSpot 4.0 Software (Cellular Technology Ltd. Europe GmbH).

### Statistical Analysis

Statistical analysis was performed using Graphpad Prism 6. Data were analyzed for statistical differences with a one-way analysis of variance (ANOVA) or unpaired *t*-test at the 5% significance level. *Post hoc* comparisons between different conditions were performed using Tukey's range test.

## Results

### Porcine Liver Resident NK Cells Display an Eomes^high^T-bet^low^ Phenotype, Are CXCR6-Positive and Lack Expression of CD49e

**Figure 1:**
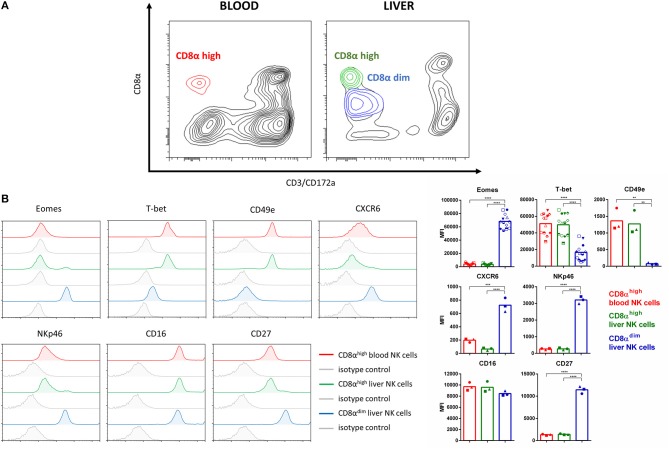
Identification of a porcine liver NK cell subpopulation that shows remarkable similarity to human lrNK cells. **(A)** Flow cytometric contour plots showing conventional NK cell populations in blood and liver and the additional liver NK population that shows lower CD8α expression. Plots show cell populations before MACS depletion and FACS sorting. A bi-exponential scale was used for the x and y-axis. **(B)** Flow cytometric histograms show the expression of Eomes, T-bet, CD49e, CXCR6, NKp46, CD16, and CD27 on conventional CD8α^high^ blood NK cells (red), conventional CD8α^high^ liver NK cells (green), and the additional CD8α^dim^ liver NK cell population (blue). Specific signals and isotype controls (gray) are shown for each marker. Graphs show the median fluorescence intensity (MFI) values for each of the markers. Bars represent the mean value, different symbols correspond to individual data points from different animals. For all markers except Eomes and T-bet, data for three different animals are shown. Since Eomes and T-bet expression was also assessed for other assays in this study as a control to check for correct phenotype of the different NK populations, individual data points for more animals (*n* = 13) are available and shown for these two markers. A logarithmic scale was used for the x-axis. Statistically significant differences are indicated with asterisks (**p < 0.01, ***p < 0.001, ****p < 0.0001).

To evaluate the possibility that the pig harbors lrNK cells, an isolation procedure of liver NK cells was performed based on previous studies that showed that mouse and human lrNK cells reside in the liver sinusoids and are enriched when the excised liver is flushed with saline (7, 8, 29, 30). Figure 1A shows that, indeed, an abundant additional NK population in the liver perfusate could be identified, which was characterized by a CD8α^dim^CD3^−^ expression pattern, compared to blood NK cells where only a CD8α^high^CD3^−^ NK population could be observed. Figure 1B shows that, in contrast to conventional blood and liver NK cells, the additional CD8α^dim^CD3^−^ liver NK cell population displays strong expression of the T-box transcription factor Eomes and low expression of T-bet, lacks detectable expression of CD49e and shows increased expression of CXCR6. This expression profile is remarkably similar to the corresponding expression profile of human lrNK cells (7, 9). In addition, compared to conventional blood and liver NK cells, the additional CD8α^dim^CD3^−^ liver NK cell population shows an increased expression of CD27 and NKp46. In line with the recent notion that in human, Eomes^high^T-bet^low^ lrNK cells can already be detected early in development (31), we found that Eomes^high^T-bet^low^ liver NK cells not only are present in mature, 6 month old pigs but also in 5-week old piglets (Supplementary Figure 1). When gating on lymphocytes (based on FSC and SSC and lack of CD172a expression), the percentage of conventional blood NK cells, conventional liver NK cells or Eomes^high^ liver NK cells were 21.0 ± 6.8%, 16.4 ± 5.2%, and 45.5 ± 13.1% for 6-month old pigs and 37.7 ± 6.7%, 38.6 + 9.1%, and 16.6 ± 1.1% for 5-week old piglets, respectively. Although this may suggest that the population of Eomes^high^ liver NK cells is higher in older pigs compared to young piglets, differences in liver perfusate preparation (e.g., differences in flushing volume as indicated in Materials and Methods) and differences in e.g., liver size and vasculature between 5-week old piglets and 6-month old pigs do not allow to draw firm conclusions in this respect.

Overall, these data demonstrate that the porcine liver harbors an NK cell population that is remarkably similar to human lrNK cells.

### Porcine Liver Eomes^high^T-bet^low^ NK Cells Lack Detectable Cytolytic Activity Against K562 Cells or PRV-Infected Cells but Do Degranulate Upon Contact With K562 Cells

**Figure 2:**
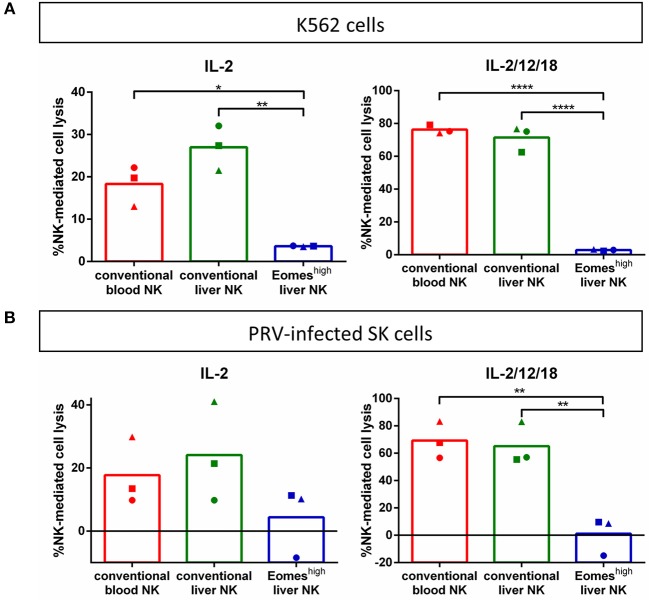
Porcine Eomes^high^ liver NK cells lack cytolytic activity against K562 cells **(A)** or PRV-infected cells **(B)**. K562 cells or PRV-infected cells were incubated with IL-2-primed or IL-2/12/18 primed primary porcine CD8α^high^ CD3^−^ blood NK cells (conventional blood NK), CD8α^high^ CD3^−^ liver NK cells (conventional liver NK) or CD8α^dim^CD3^−^ liver resident NK cells (Eomes^high^ liver NK) at a target:effector ratio of 1:20 for 4 h at 37°C. Viability of target cells was assessed by propidium iodide staining and flow cytometric analysis, and the percentage of NK cell-mediated lysis was calculated. Graphs shows percentage of NK cell-mediated lysis, showing individual data points for three different animals, and the mean value indicated by the bar. Statistically significant differences are indicated with asterisks (*p < 0.05,**p < 0.01, ****p < 0.0001).

To assess the ability of porcine Eomes^high^T-bet^low^ liver NK cells to kill NK-susceptible target cells, cytolytic assays were performed using CFSE-labeled K562 cells. K562 are human leukemia cells that show high susceptibility toward killing by NK cells from a variety of different species, including porcine blood NK cells (13, 17, 32). K562 cells were co-incubated with freshly isolated IL-2- or IL-2/IL-12/IL-18-primed porcine conventional blood NK cells, conventional liver NK cells or Eomes^high^T-bet^low^ lrNK cells and subsequently assessed for NK cell-mediated cytotoxicity by flow cytometry. Figure 2A shows that, in contrast to conventional blood and liver Eomes^low^T-bet^high^ NK cells, the porcine liver Eomes^high^T-bet^low^ NK cell population apparently lacks the ability to kill K562 cells. To determine whether this lack of detectable cytolytic activity was due to the nature of the target cells, additional cytolytic assays were performed using PRV- or mock-infected SK cells as target cells. We showed earlier that PRV-infected SK cells, but not mock-infected SK cells, are susceptible to the cytolytic activity of porcine blood NK cells (12, 25–27). However, also against PRV-infected target cells, no detectable cytolytic activity could be observed when using porcine liver Eomes^high^T-bet^low^ NK cells as effector cells (Figure 2B).

**Figure 3:**
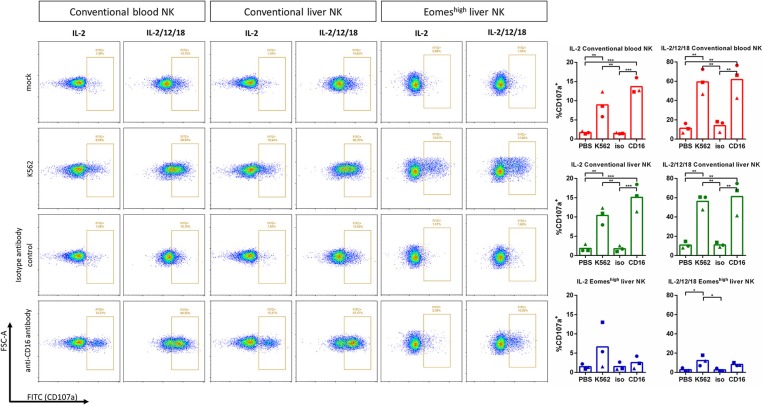
Degranulation capacity of Eomes^high^ liver NK cells upon contact with K562 cells or CD16 crosslinking. IL-2 or IL-2/12/18 primed porcine CD8α^high^ CD3^−^ blood NK cells (conventional blood NK), CD8α^high^ CD3^−^ liver NK cells (conventional liver NK) or CD8α^dim^CD3^−^ liver resident NK cells (Eomes^high^ liver NK) were incubated at a target:effector ratio of 1:1 with K562 cells (K562) or added to anti-CD16 antibody coated plates for 4 h at 37°C. Irrelevant isotype-matched antibody coated plates (isotype antibody control) and PBS (mock) served as negative control. Degranulation was assessed by measuring the expression of CD107a on the cell surface by flow cytometry. Graphs show the percentage of CD107a^+^ cells, showing individual data points for three different animals, and the mean value indicated by the bar. A logarithmic scale was used for the x-axis. Statistically significant differences are indicated with asterisks (*p < 0.05, **p < 0.01, ***p < 0.001).

As a more sensitive method to detect cytolytic activity, degranulation assays were performed based on CD107a detection (18), using either K562 cells or anti-CD16 antibody-coated plates as stimulant for degranulation. Figure 3 shows that, although much less pronounced than in conventional blood and liver NK cells, K562 cells triggered degranulation in Eomes^high^T-bet^low^ liver NK cells. Anti-CD16 antibody also reproducibly triggered increased degranulation, although this increase did not reach statistical significance.

### Porcine Liver Eomes^high^T-bet^low^ NK Cells Are Able to Produce IFN-γ Upon Cytokine Stimulation

**Figure 4:**
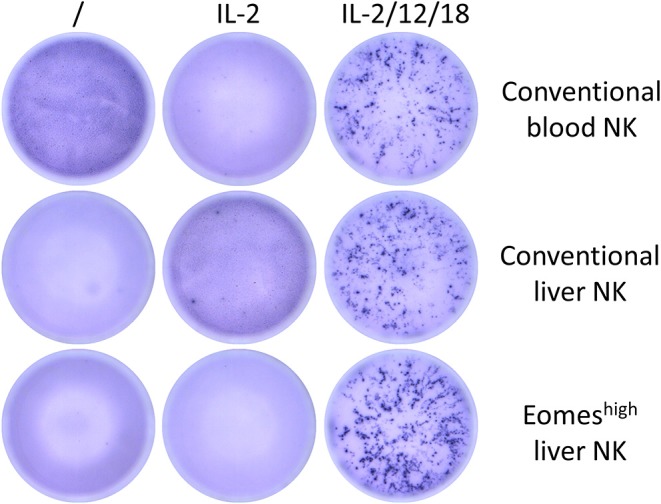
IFN-γ production of conventional blood and liver NK cells or Eomes^high^ liver resident NK cells upon stimulation with IL-2/12/18. After NK isolation, 1.25 × 10^4^ NK cells/well were cultivated without stimulation or with different cytokine mixes for 20 or 38 h and analyzed by ELISpot for their IFN-γ production. Picture shows data for one representative animal (analyzed at 20 h post cytokine addition) out of five animals analyzed in total.

Next, the ability of Eomes^high^T-bet^low^ liver NK cells to produce IFN-γ was evaluated. Cytokines (IL-2, IL-12, and IL-18) were added as stimuli to sorted porcine blood and liver NK cells. Figure 4 shows that all three types of NK cell populations were able to produce IFN-γ upon IL-2/12/18 stimulation. Hence, Eomes^high^T-bet^low^ NK cells are able to produce IFN-γ upon IL-2/12/18 stimulation.

### Is There Plasticity Between Porcine Conventional Eomes^low^ Blood NK Cells and Eomes^high^ Liver NK Cells?

In human and mouse, some degree of cytokine-induced plasticity between blood NK cells and lrNK cells has been suggested, including IL-12/IL-15-induced expression of CXCR6 on murine blood NK cells and Eomes in human blood NK cells (8, 33). Therefore, we wanted to investigate whether conventional porcine Eomes^low^ blood NK cells can be stimulated to differentiate toward a phenotypic profile that corresponds to some extent to that of Eomes^high^ liver NK cells.

**Figure 5:**
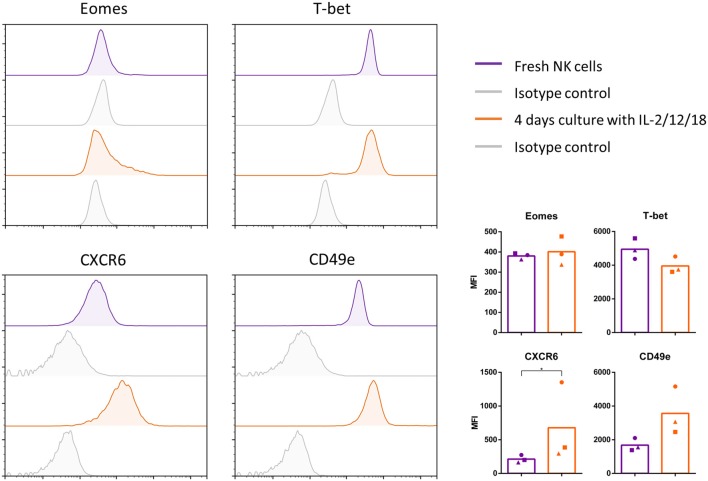
Conventional Eomes^low^ blood NK cells show some degree of plasticity with regard to phenotypic expression marker profile. NK cells were sorted from blood, examined for their expression of Eomes, T-bet, CXCR6, and CD49e (purple) and cultured for 4 days in the presence of IL-2/12/18 (orange). Afterwards, cells were examined for their expression of Eomes, T-bet, CXCR6, and CD49e. Flow cytometric histrograms of the signals and isotype controls (gray) are shown. Graphs show the median fluorescence intensity (MFI), showing individual data points for three different animals, and the mean value indicated by the bar. A logarithmic scale was used for the x-axis. Statistically significant differences are indicated with an asterisk (*p < 0.05).

Figure 5 shows that stimulation of porcine blood NK cells with IL-2/12/18 leads to a significant upregulation of CXCR6 expression and shows a slight, but non-statistically significant decrease in T-bet expression. Treatment of blood NK cells with the IL-2/12/18 cytokine mix did not lead to significant changes in Eomes expression or a decrease in CD49e expression. Rather, a reproducible but statistically non-significant increase in CD49e expression could be observed. Similar results were obtained upon addition of IL-15 to the cytokine mix (IL-2/12/15/18 treatment, data not shown).

Still, the increase in CXCR6 expression suggests that some degree of cytokine-induced plasticity may exist between NK cell subpopulations in pig.

## Discussion

Over the past decade, there has been an increasing interest in long-lived lrNK cells. Although lrNK cells have been identified in man and mouse, and although some similarities exist between lrNK cell populations in both species (e.g. both are CXCR6^+^), this cell population shows remarkable species-specific differences in T-bet transcription factor expression profile (Eomes^high^T-bet^low^ in human lrNK cells and *vice versa* in mouse lrNK cells) and in cell surface protein expression (e.g., mouse lrNK cells are CD49a^+^ whereas human lrNK cells are CD49e^−^). This obviously complicates interpretation and extrapolation of mouse experiment data on this particular NK cell population. In the current paper, we demonstrate that the pig may represent a particularly attractive model species to study the function and characteristics of lrNK cells, as we identify a porcine liver NK cell population that shows striking phenotypic similarity with human lrNK cells, i.e., Eomes^high^T-bet^low^, CD49e^−^ and CXCR6^+^.

Our data combined with earlier data on mouse and human lrNK cells point out that, across species, CXCR6 expression is a common hallmark of these cells. The expression of CXCR6 presumably contributes to homing to and retention of these NK cells in the liver sinusoids, where the CXCR6 ligand CXCL16 is produced (34).

Further in line with studies in man, we found that porcine Eomes^high^ T-bet^low^ NK cells were (almost) completely absent from blood (7–9, 35). Human Eomes^high^ NK cells do not appear to exit the liver and have been reported to persist in the liver for up to at least 13 years (8). Human Eomes^low^ conventional NK cells (cNK) on the other hand can circulate freely and can also be found in the liver, which is in line with our results (8). Upon *in vitro* cultivation with cytokines (e.g., IL-12/15), human Eomes^low^ conventional blood and liver NK cells were found to upregulate Eomes (8). In addition, IL-12/15 stimulation has been described to stimulate CXCR6 expression on human conventional blood NK cells (33). To some extent in line with these results, we found that porcine conventional blood NK cells upregulated CXCR6 expression upon IL-2/12/(15)/18 stimulation. IL-2/12/18 cytokine stimulation of porcine blood NK cells also resulted in upregulation of NKp46 (data not shown), correlating with the higher NKp46 expression observed in porcine liver Eomes^high^ NK cells. This result is in line with earlier observations that a NKp46^−^ NK cell subpopulation in porcine blood became NKp46^+^ upon IL-2/12/18 stimulation (17). Although this may suggest that both in man and pig, there may be some plasticity between conventional blood and liver NK cells (8), addition of IL-2/12/18 did not substantially affect Eomes expression levels.

Despite indications for some cytokine-dependent plasticity in phenotypic marker expression profile between porcine conventional blood NK cells and liver NK cells, the cytokine environment used in our assays did not obviously convert the blood NK cell marker profile to that of liver Eomes^high^ NK cells. Indeed, IL-2/12/18 stimulation of blood NK cells resulted in an upregulation of CD49e, whereas this marker is not/less expressed on the surface of lrNK cells in both pig and man. One speculative explanation may be that upon (cytokine-driven) homing of NK cells to the liver, e.g., under the influence of CXCR6-CXCL16, the liver micro-environment may provide particular cytokines and growth factors that may lead to further differentiation of these incoming NK cells to lrNK cells. The presence of CXCR6^+^ Eomes^low^ NK cells in blood of humans and pigs may support this hypothesis, where the liver micro-environment may contribute to further differentiation of lrNK cells, including increased expression of Eomes (9, 36). It will be interesting to address in future studies whether elements of the liver micro-environment, including e.g., factors expressed by hepatocytes and Kupffer cells, may contribute to the phenotypic and functional characteristics of lrNK cells.

We found that porcine lrNK cells showed some degranulation upon contact with NK-susceptible K562 target cells or, albeit not statistically significant, upon CD16 crosslinking, but were unable to kill either K562 cells or PRV-infected SK cells in standard assays that resulted in substantial lysis by conventional blood and liver NK cells (12, 25, 26). Although this suggests that porcine Eomes^high^ NK cells show poor cytolytic capacity, an alternative or additional possibility may be that Eomes^high^ NK cells may require specific priming factors to increase their cytolytic potential. In the current study and many other studies (12, 25, 26), IL-2 is typically used to prime primary NK cells for cytolytic assays. Of note, by flow cytometry, we could demonstrate that porcine lrNK cells show a decreased surface expression of the alpha-chain (CD25) of the high-affinity IL-2 receptor (Supplementary Figure 2). Hence, in future studies, it will be interesting to test additional stimuli and NK-susceptible target cells to fully understand the cytolytic capacity of porcine lrNK cells and compare it to that of conventional NK cells.

Nonetheless, the reduced cytolytic activity that we observed for porcine liver Eomes^high^ NK cells is not surprising as it is in line with some reports on human lrNK cells. Indeed, human lrNK cells have been reported to also display weak cytolytic activity, which has been attributed to their limited expression of inhibitory killer Ig-like receptors (31, 37). IFN-γ ELISpot assays showed that porcine Eomes^high^ NK cells have the capacity to produce IFN-γ upon cytokine stimulation. Human lrNK cells have also been reported to produce IFN-γ upon stimulation, although the degree of IFN-γ production appears to depend on the particular stimulus (31, 38).

Although several reports have addressed the phenotypic characterization of the human lrNK cell population, many questions remain regarding their functional significance (9, 33, 35). The conservation of these cells between man and pig and their presence in both species early in development as well as later in life (33, current report), contribute to the notion that these cells may play an important role in the (hepatic) immune system. In line with the observed weak cytolytic potential and their longevity, this NK cell subset has been hypothesized to contribute to immune tolerance and/or memory responses (6, 39–42). The current findings emphasize that porcine liver Eomes^high^ NK cells represent a unique and complementary model to address these particular aspects of NK cell biology and provide an important tool to assess the importance of lrNK cells in health and (liver) disease and their potential role in prophylaxis and therapy.

## Data Availability Statement

The datasets generated for this study are available on request to the corresponding author.

## Ethics Statement

The blood sampling and euthanasia procedures were approved by the Ethical Committee of the Faculty of Veterinary Medicine (EC2013/62 and EC2017/121).

## Author Contributions

SDP, SD, and LH performed the experiments. SDP, SD, and HF designed the research, made the figures, wrote the manuscript, and analyzed the results.

### Conflict of Interest

The authors declare that the research was conducted in the absence of any commercial or financial relationships that could be construed as a potential conflict of interest.
