# Supplementary material for: Identification of a Porcine Liver EomeshighT-betlow NK Cell Subset That Resembles Human Liver Resident NK Cells
Source: Front Immunol. 2019 Oct 31;10:2561. doi: 10.3389/fimmu.2019.02561 (PMC6836759; doi:10.3389/fimmu.2019.02561)
Supplement: Supplementary file 1 [file Data_Sheet_1.PDF]

Supplementary Material

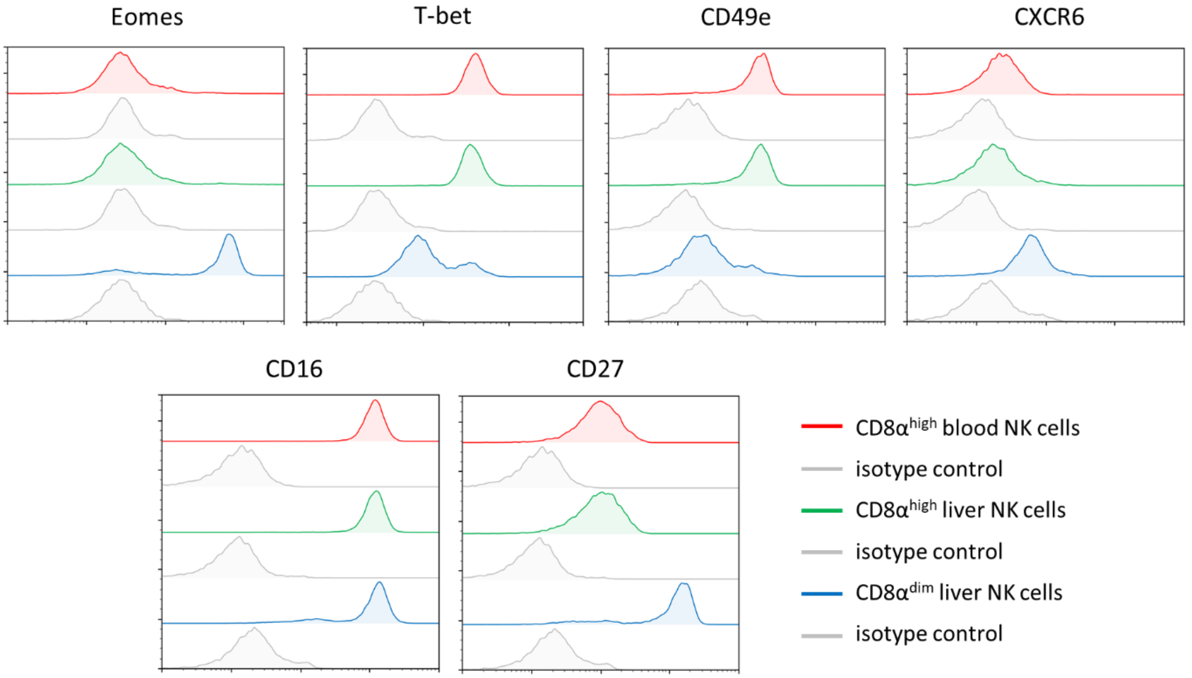

Supplementary Figure 1: Identification of a porcine liver NK cell subpopulation that shows remarkable similarity to human lrNK cells. Flow cytometric histograms show the expression of Eomes, T-Bet, CD49e, CXCR6, CD16 and CD27 on conventional CD8α^{high} blood NK cells (red), conventional CD8α^{high} liver NK cells (green) and the additional CD8α^{dim} liver NK cell population (blue) of 5-week old piglets. Specific signals and isotype controls (grey) are shown for each marker. A logarithmic scale was used for the x-axis. Histograms show the results of one representative animal out of 3 independent repeats.

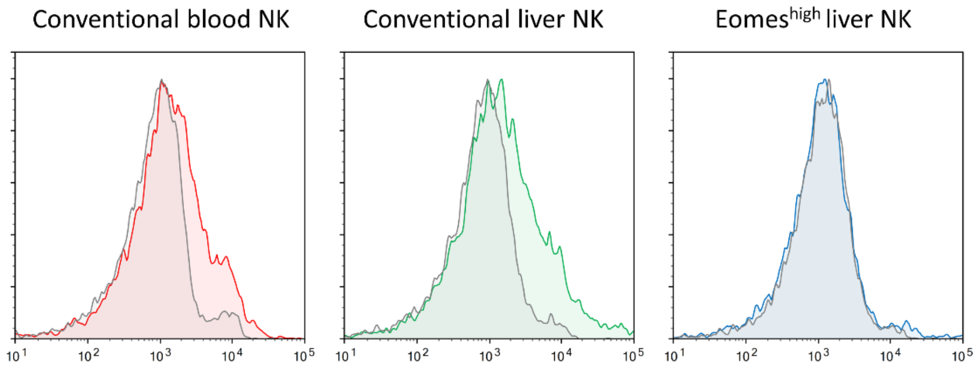

Supplementary Figure 2: CD25 expression on blood and liver NK cells. Flow cytometric histograms show the expression of CD25 on conventional blood NK cells (red), conventional liver NK cells (green) and the Eomes^{high} liver NK cells (blue). Specific signals and isotype controls (grey) are shown for each marker. A logarithmic scale was used for the x-axis.
